# Thrombospondin-2 acts as a bridge between tumor extracellular matrix and immune infiltration in pancreatic and stomach adenocarcinomas: an integrative pan-cancer analysis

**DOI:** 10.1186/s12935-022-02622-x

**Published:** 2022-06-14

**Authors:** Xingchen Liao, Wei Wang, Baoping Yu, Shiyun Tan

**Affiliations:** 1 Department of Gastroenterology, Renmin Hospital of Wuhan University, Wuhan, 430060 China; 2 Department of Hepatobiliary Surgery, East Hospital, Renmin Hospital of Wuhan University, Wuhan, Hubei China; 3 Key Laboratory of Hubei Province for Digestive Disease, Renmin Hospital of Wuhan University, Wuhan, 430060 China; 4 Central Laboratory, Renmin Hospital of Wuhan University, Wuhan, 430060 China

**Keywords:** Thrombospondin-2, Pan-cancer analysis, Extracellular matrix, Immune infiltration, Tumor microenvironment, Prognosis, Biomarker

## Abstract

**Background:**

Thrombospondin-2 (THBS2) is a versatile glycoprotein that regulates numerous biological functions, including the apoptosis-proliferation balance in endothelial cells, and it has been linked to tumor angiogenesis. However, the exact role of THBS2 in human cancer remains unknown. This study aimed to determine THBS2 expression in a pan-cancer analysis and its association with pan-cancer prognosis and to further identify its possible roles in tumor immunity and the extracellular matrix (ECM).

**Methods:**

Data on THBS2 expression in cancers and normal tissues were downloaded from the Genotype-Tissue Expression portal and UCSC Xena visual exploration tool and analyzed using the ONCOMINE database, Perl programming language, and Gene Expression Profiling and Interactive Analyses vision 2 webserver. In addition, survival prognosis was analyzed using the survival, survminer, limma, and forestplot packages in R v. 4.0.3.Immune and matrix components were also analyzed using R v. 4.0.3. Most importantly, we partially validated the role and mechanism of THBS2 in pancreatic and gastric cancers in vitro using PANC1 and BGC-823 cell lines.

**Results:**

THBS2 was significantly overexpressed in 17 of the 33 investigated cancers and linked to a poor prognosis in pan-cancer survival analysis. High THBS2 expression was an independent unfavorable prognostic factor in kidney renal papillary cell, mesothelioma, and stomach and pancreatic adenocarcinomas. Immune infiltration and THBS2 expression were also related. THBS2 expression has been linked to immune and stromal scores and immune checkpoint markers in various cancers. The protein–protein interaction network revealed that THBS2 is associated with multiple ECM and immune proteins. THBS2 knockdown decreased the expression of CD47 and matrix metallopeptidase 2 (MMP-2) as well as the proliferation, migration, and invasion of PANC1 and BGC-823 cells in vitro.

**Conclusions:**

Our findings suggested that THBS2 might promote cancer progression by remodeling the tumor microenvironment, affecting CD47-mediated signaling pathways, activating the pro-tumor functions of a disintegrin and metalloproteinase with thrombospondin motifs, and enhancing MMP-2 expression. Furthermore, it functions as a bridge between the ECM and immune infiltration in cancer and serves as a potential prognostic biomarker for several cancers, especially pancreatic and gastric adenocarcinomas.

**Supplementary Information:**

The online version contains supplementary material available at 10.1186/s12935-022-02622-x.

## Background

Thrombospondin-2 (THBS2) is an extracellular matrix (ECM) protein belonging to the THBS family. Stromal fibroblasts and endothelial and immune cells secrete THBS2, which functions in cell migration, angiogenesis, apoptosis, and cytoskeletal regulation [1]. The significantly elevated expression of THBS2 in various cancer types, including pancreatic and gastric cancers, is associated with their diagnosis, stage, and prognosis. Andreas et al. [2] found that early pancreatic cancer can be screened using serum markers, including THBS2. Li [3] found that combining THBS2 with CA19-9 improved the detection of early gastric cancer. THBS2 is also a potential prognostic biomarker of colorectal, pancreatic, and gastric cancers [4].

The interplay between tumors and the immune system is complex. The tumor microenvironment (TME) is crucial for tumorigenesis and progression in human cancers and has recently become a popular topic in tumor research. In addition to cellular components, the TME contains non-cellular components dominated by infiltrating immune cells [5] comprising tumor-associated macrophages (TAMs), B cells, CD4 + and CD8 + T cells, and neutrophils. In addition to mediating the immune escape of tumor cells, tumor angiogenesis, and metastasis [6–8], TAMs can affect the prognosis of patients and the effect of immunotherapy [9, 10]. Compared with the limitations of traditional anticancer therapies, alternative immunotherapy has achieved good results in various cancers [11–13]. However, the relevance of THBS2 in tumor immunity and the underlying mechanisms remain unclear.

The aim of this study was to reveal the relationship between THSB2 expression and prognosis of tumor patients, and the interaction of THBS2 expression with immune cell infiltration and extracellular matrix proteins, providing further insights into cancer prevention and treatment targets.

## Methods

### Cancer types assessed

Table 1 shows the 33 types of cancer that were assessed in this study.

**Table 1:** Cancer types assessed in the present study

Abbreviations and full names of cancer types			
ACC	Adrenocortical carcinoma	LUSC	Lung squamous cell carcinoma
BLCA	Bladder Urothelial Carcinoma	MESO	Mesothelioma
BRCA	Breast invasive carcinoma	OV	Ovarian serous cystadenocarcinoma
CESC	Cervical squamous cell carcinoma and endocervical adenocarcinoma	PAAD	Pancreatic adenocarcinoma
CHOL	Cholangiocarcinoma	PCPG	Pheochromocytoma and Paraganglioma
COAD	Colon adenocarcinoma	PRAD	Prostate adenocarcinoma
DLBC	Lymphoid Neoplasm Diffuse Large B-cell Lymphoma	READ	Rectum adenocarcinoma
ESCA	Esophageal carcinoma	SARC	Sarcoma
GBM	Glioblastoma multiforme	SKCM	Skin Cutaneous Melanoma
HNSC	Head and Neck squamous cell carcinoma	STAD	Stomach adenocarcinoma
KICH	Kidney Chromophobe	TGCT	Testicular Germ Cell Tumors
KIRC	Kidney renal clear cell carcinoma	THCA	Thyroid carcinoma
KIRP	Kidney renal papillary cell carcinoma	THYM	Thymoma
LAML	Acute Myeloid Leukemia	UCEC	Uterine Corpus Endometrial Carcinoma
LGG	Brain Lower Grade Glioma	UCS	Uterine Carcinosarcoma
LIHC	Liver hepatocellular carcinoma	UVM	Uveal Melanoma
LUAD	Lung adenocarcinoma		

### Gene expression analysis

The mRNA expression of THBS2 in different cancers was analyzed using the ONCOMINE database (www.oncomine.org). The THBS2 expression data for different normal tissues and cancers was acquired from the Gene Expression Profiling and Interactive Analyses vision 2 (GEPIA2) webserver (http://gepia2.cancer-pku.cn/) and Genotype-Tissue Expression (GTEx) database.

### Pan-cancer dataset source and processing

Gene-expression data and full clinical annotation of the 33 cancer datasets were obtained from UCSC Xena (https://xenabrowser.net.). We then used Perl software (version 5.34.0) (https://www.perl.org/) to sort out the matrix data of gene expression values.

### Survival prognosis

Survival (overall survival [OS], disease-specific survival [DSS], disease-free interval [DFI], and progression-free interval [PFI]) and prognosis were analyzed using the survival, survminer, and forestplot packages in R v. 4.0.3 and the Bioconductor package limma in R. RNA-sequencing expression (level 3) profiles and corresponding clinical information for cancers were downloaded from The Cancer Genome Atlas (TCGA) dataset (https://portal.gdc.com). Univariate and multivariate Cox regression analyses were performed, and forest plots were used to show the *P*-value, hazard ratio (HR), and 95% class interval of each variable using the ‘forestplot’ R package.

### Immune infiltration

We analyzed immune and matrix components in the TME using the estimate package in R (version 4.0.3), as well as ImmuneScore, StromalScore, and EstimatScore. The associations between THBS2 and ImmuneScore and StromalScore in the 33 types of cancers were also analyzed. In addition, we calculated the putative proportions of immune cells from gene expression profiles using the online analytical platform CIBERSORT (https://cibersort.stanford.edu/) [14]. Finally, we estimated the relationship between THBS2 and the relative abundance of tumor-infiltrating immune cells in the 33 cancer types using a reference set with 22 sorted immune cell subtypes (LM22) and CIBERSORT.

### Cell culture

We cultured human stomach cancer (BGC-823) and human pancreatic cancer (PANC1) cells (China Center for Type Culture Collection, Wuhan, China) in RPMI 1640 medium and cultured them in Dulbecco Modified Eagle medium. The cells were routinely maintained and supplemented with 10% fetal bovine serum and 1% peni-cillin/streptomycin (C0222; Beyotime Biotechnology) at 37 °C with 5% CO2.

### Transfection

Cells were transfected with small interfering (si) THBS2 mRNA or negative control (NC) siRNA, which were designed and produced (GenePharma, Shanghai, China) using the following forward and reverse (5′ 3′) primers:

THBS2-SiRNA: GUUUGCUUCAGAACGUCCATT and UGGACGUUCUGAAGCAAACTT.

NC: UUCUUCGAACGUGUCACGUTT and ACGUGACAUGUUCGGAGAATT.

Thereafter, cancer cells were transfected with siTHBS2 or NC using Lipofectamine2000 (Thermo Fisher Scientific, Waltham, MA, USA).

### Cell migration, invasion, and proliferation assay

We evaluated the migration and proliferative capacity of PANC1 and BGC-823 cells using CCK-8, transwell, and wound healing assays.

### Western blotting

Total protein from PANC1 and BGC-823 cells extracted using radioimmunoprecipitation assay buffer was resolved by gel electrophoresis and then electroblotted onto polyvinylidene difluoride membranes. Subsequently, non-specific antigen binding was blocked using 5% skim milk, and the membranes were then incubated at 4 °C with gentle shaking overnight with the following primary antibodies: 1:500-diluted THBS2 (A8561) and 1:1,000-diluted CD47 (A1838; both from ABclonal Technology, Woburn, MA, USA), 1:100-diluted matrix metalloproteinase (MMP)-2 (sc-13095; Santa Cruz Biotechnology, Dallas TX, USA), or 1:5,000-diluted GAPDH (60,004–1-Ig; ProteinTech Group, Rosemont, IL, USA). Thereafter, the membranes were incubated on the following day with 1:5,000-diluted secondary antibodies (SA00001-1 and SA00001-2; ProteinTech Group).

### Quantitative reverse-transcription PCR

Quantitative reverse-transcription polymerase chain reaction (qRT-PCR) was conducted using the StepOne™ PCR Detection System (Life Technologies, Carlsbad, CA, USA) and the forward and reverse (5′ 3′) primers:

GAPDH: CATCATCCCTGCCTCTACTGG and GTGGGTGTCGCTGTTGAAGTC;

THBS2: TCCTGCTGGCTCTGTGGGTGT; TGTGTTCTCACTGATGGCGT.

### Statistical analysis

Differences between normal and cancer tissues were compared using Student’s *t*-tests. Associations between THBS2 expression and patient survival were investigated using univariate survival analysis and Kaplan–Meier curves. Values with *P* < 0.05 were considered statistically significant. The statistical significance of the in vitro data was determined using GraphPad Prism 7 (GraphPad Software Inc., San Diego, CA, USA).

## Results

### Pan-cancer THBS2 mRNA expression

**Fig. 1:**
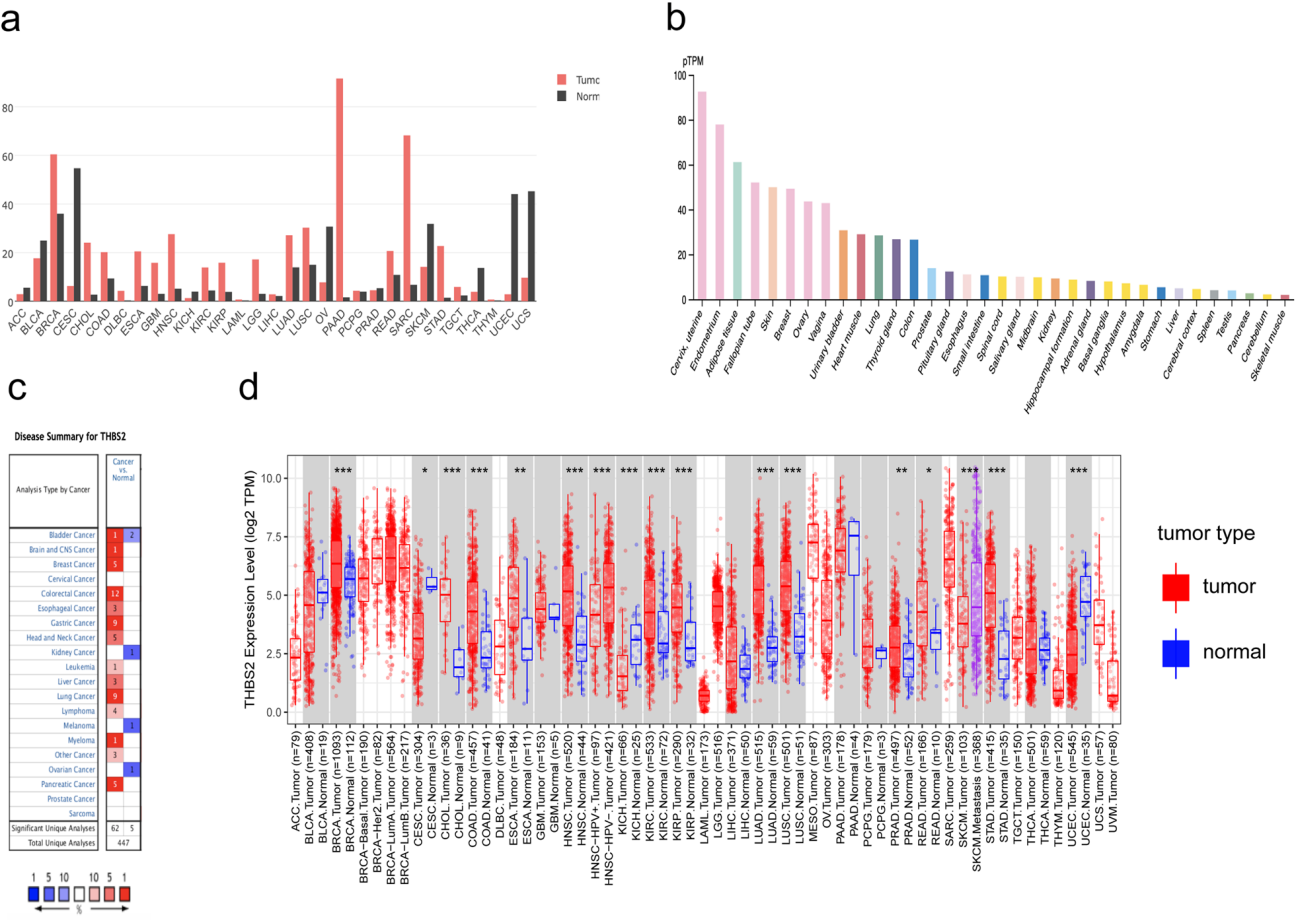
Expression of THBS2 mRNA in pan-cancer analysis. **a** Differential expression of THBS2 in cancers and normal tissues determined using GEPIA2. **b** THBS2 expression in 34 normal tissues determined using the GTEx database. **c** Comparison of THBS2 expression between normal and cancer tissues in ONCOMINE. **d** Expression of THBS2 in various cancer types according to TIMER2

We evaluated THBS2 expression in 34 normal tissues from the GTEx database. The results showed that THBS2 was significantly higher in the cervix, uterus, endometrium, and adipose tissue and significantly lower in the skeletal muscle, cerebellum, pancreas, and stomach (Fig. 1b). We further analyzed the mRNA expression of THBS2 in the ONCOMINE database to explore pan-cancer THBS2 expression. The findings revealed that cancer groups, such as bladder, colorectal, breast, esophageal, gastric, leukemia, liver, lung, lymphoma, myeloma, ovarian, pancreatic, and brain and central nervous system cancers, as well as head and neck cancer, showed higher THBS2 expression than that of normal groups (Fig. 1c). We used TIMER2 to analyze RNA sequencing data from the TCGA database and evaluate pan-cancer THBS2 expression. The differential expression of THBS2 in tumor and normal tissues is shown in Fig. 1d. THBS2 expression was significantly lower in CESC, KICH, and UCEC than that in normal tissues. However, in BRCA, COAD, CHOL, ESCA, KIRC, KIRP, LUAD, LUSC, PRAD, PEAD, and STAD, THBS2 expression was significantly higher than that in normal tissues. We then analyzed pan-cancer THBS2 expression and that in normal tissues using GEPIA2, which contains data for 31 tumor tissues from the TCGA database, as well as that of their corresponding normal tissues in the GTEx database. The results indicated that THBS2 expression was significantly higher in 17 cancers, mainly in BRCA, PAAD, and SARC, and significantly lower in 14 cancers, especially CESC, UCEC, and UCS (Fig. 1a). These findings reveal that THBS2 is aberrantly expressed in various cancers.

### Pan-cancer prognostic value of THBS2

**Fig. 2:**
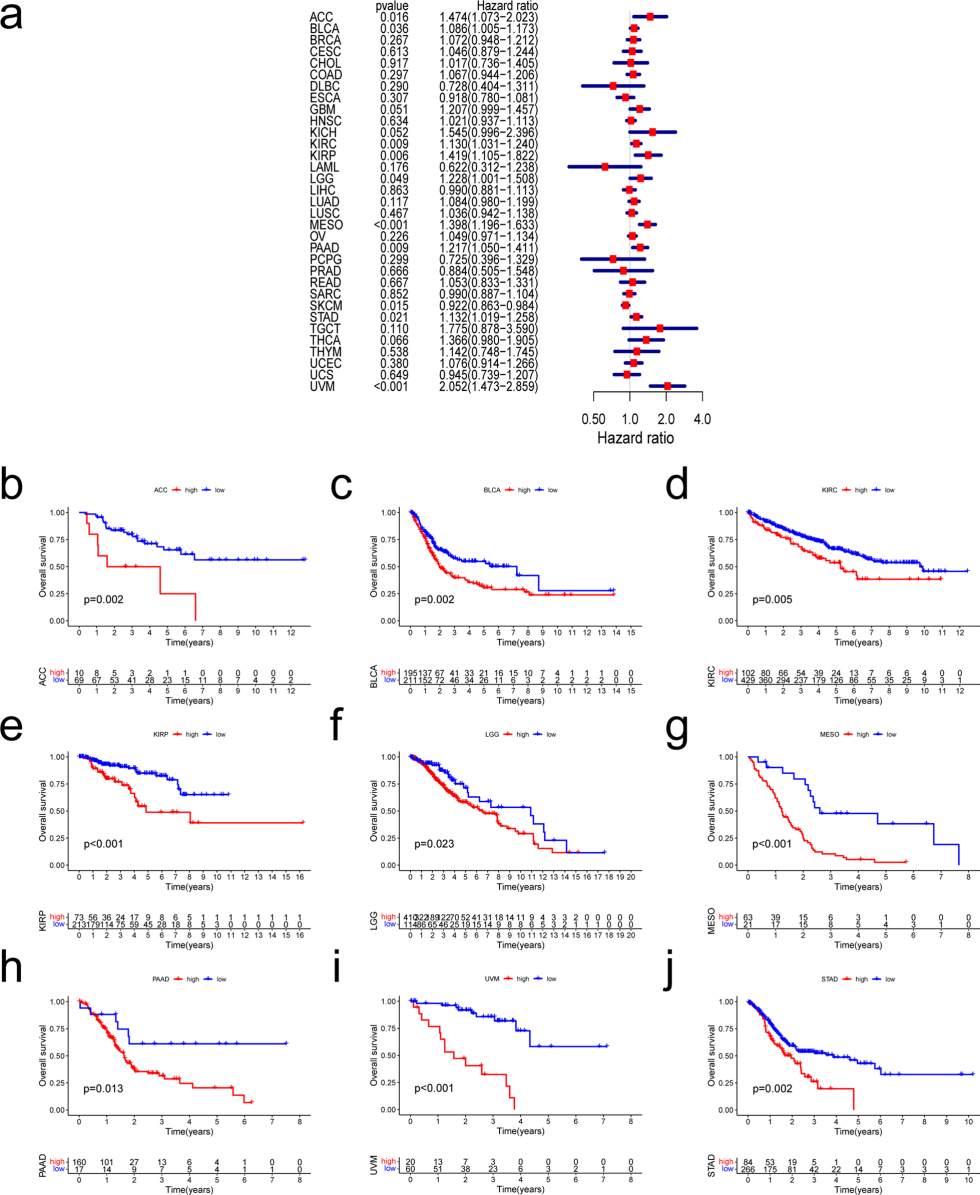
Correlations between THBS2 expression and overall survival. **a** Relationship between hazard ratios and THBS2 expression in the 33 types of cancer. **b**–**j** Kaplan–Meier curves showing relationships between differential expression of THBS2 and overall survival of patients with adrenocortical carcinoma (ACC), bladder urothelial carcinoma (BLCA), kidney renal clear cell carcinoma (KIRC), kidney renal papillary cell carcinoma (KIRP), brain lower-grade glioma (LGG), mesothelioma (MESO), pancreatic adenocarcinoma (PAAD), stomach adenocarcinoma (STAD), skin cutaneous melanoma (SKCM), and uveal melanoma (UVM)

**Fig. 3:**
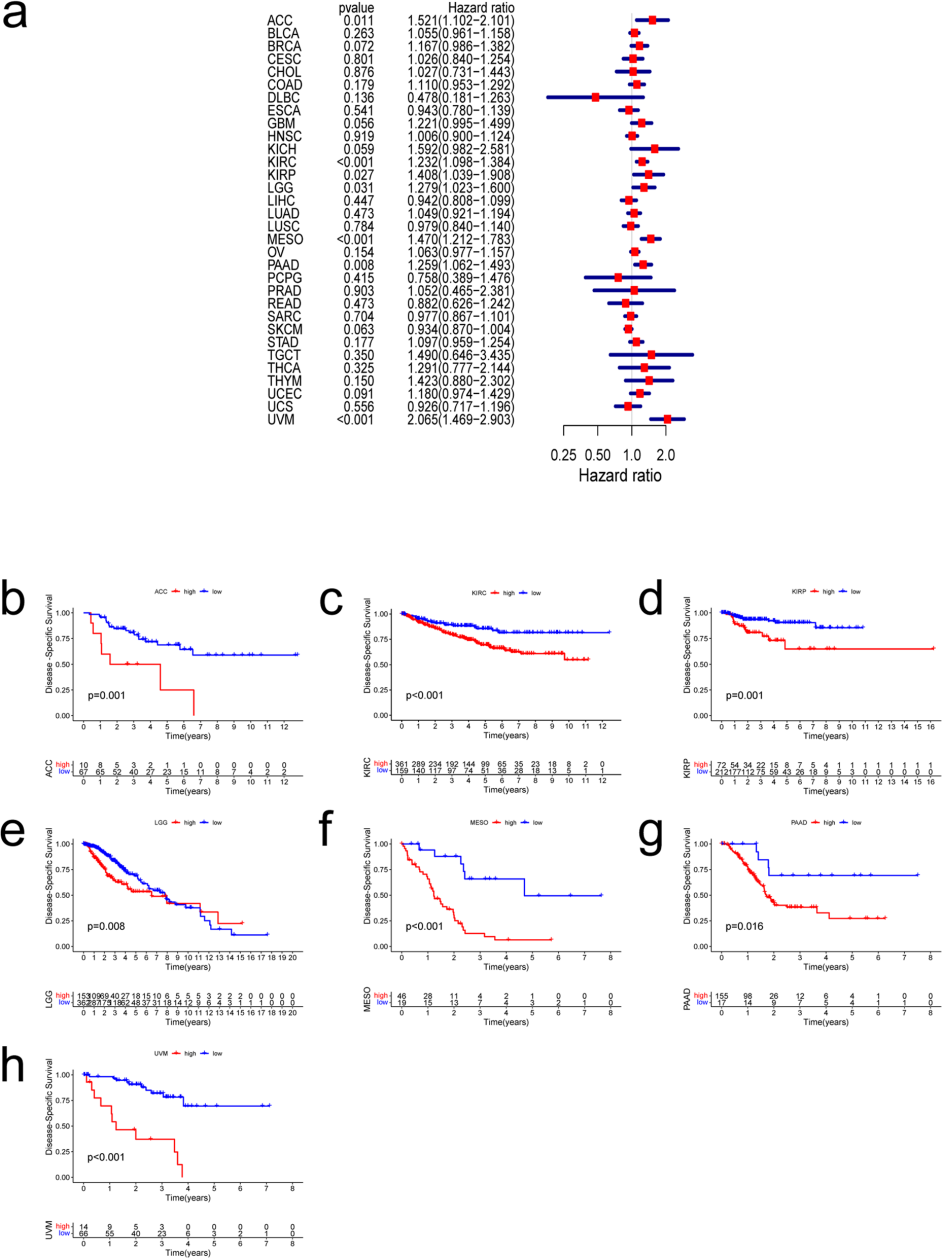
Correlation between THBS2 expression and disease-specific survival. **a** Relationships between hazard ratios and THBS2 expression in 32 cancers. **b**–**h** Kaplan–Meier curves showing relationships between differential expression of THBS2 and disease-specific survival of adrenocortical carcinoma (ACC), kidney renal clear cell carcinoma (KIRC), kidney renal papillary cell carcinoma (KIRP), brain lower-grade glioma (LGG), mesothelioma (MESO), pancreatic adenocarcinoma (PAAD), and uveal melanoma (UVM)

**Fig. 4:**
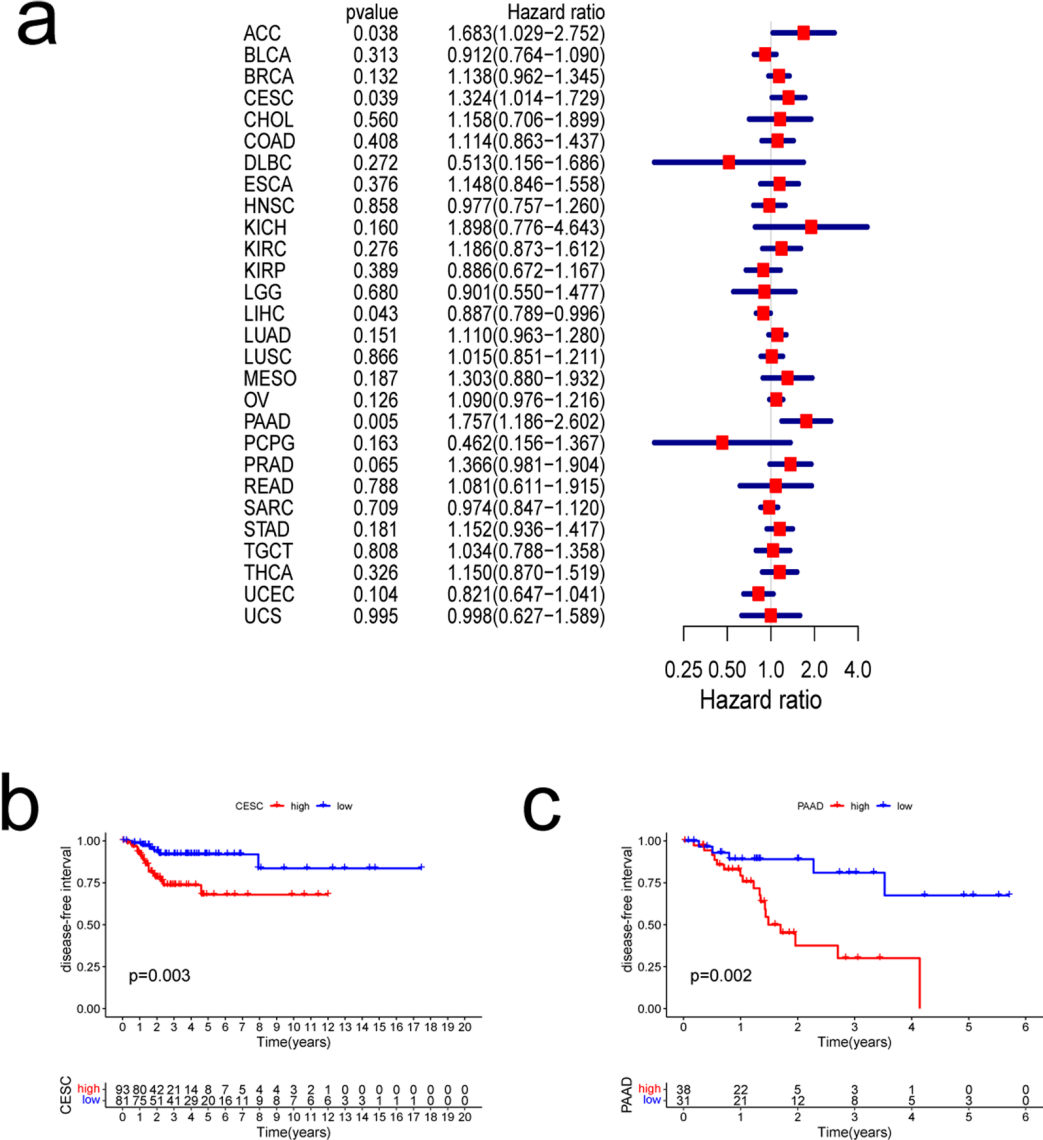
Correlations between THBS2 expression and disease-free interval. **a** Relationships between hazard ratios and THBS2 expression in 28 types of cancer. **b**, **c** Kaplan–Meier curves showing relationships between differential expression of THBS2 and disease-free intervals in cervical squamous cell carcinoma and endocervical (CESC) and pancreatic adenocarcinomas (PAAD)

**Fig. 5:**
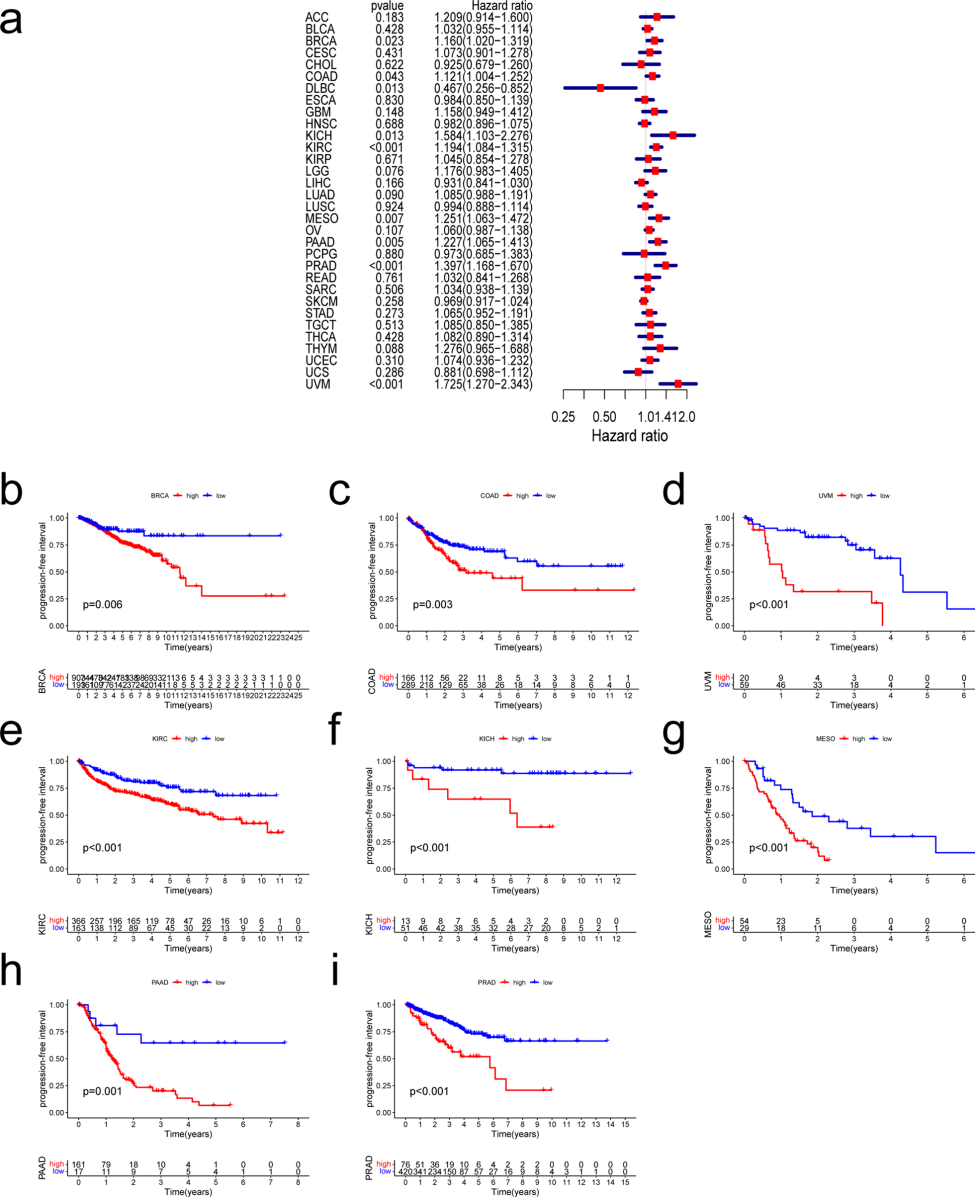
Correlations between THBS2 expression and platinum-free interval. **a** Relationships between hazard ratios and THBS2 expression in 32 types of cancer. **b**–**i** Kaplan–Meier curves showing relationships between differential expression of THBS2 and platinum-free intervals in breast invasive carcinoma (BRCA), colon adenocarcinoma (COAD), kidney chromophobe (KICH), kidney renal clear cell carcinoma (KIRC), mesothelioma (MESO), pancreatic adenocarcinomas (PAAD), prostate adenocarcinoma (PRAD), and uveal melanoma (UVM)

**Fig. 6:**
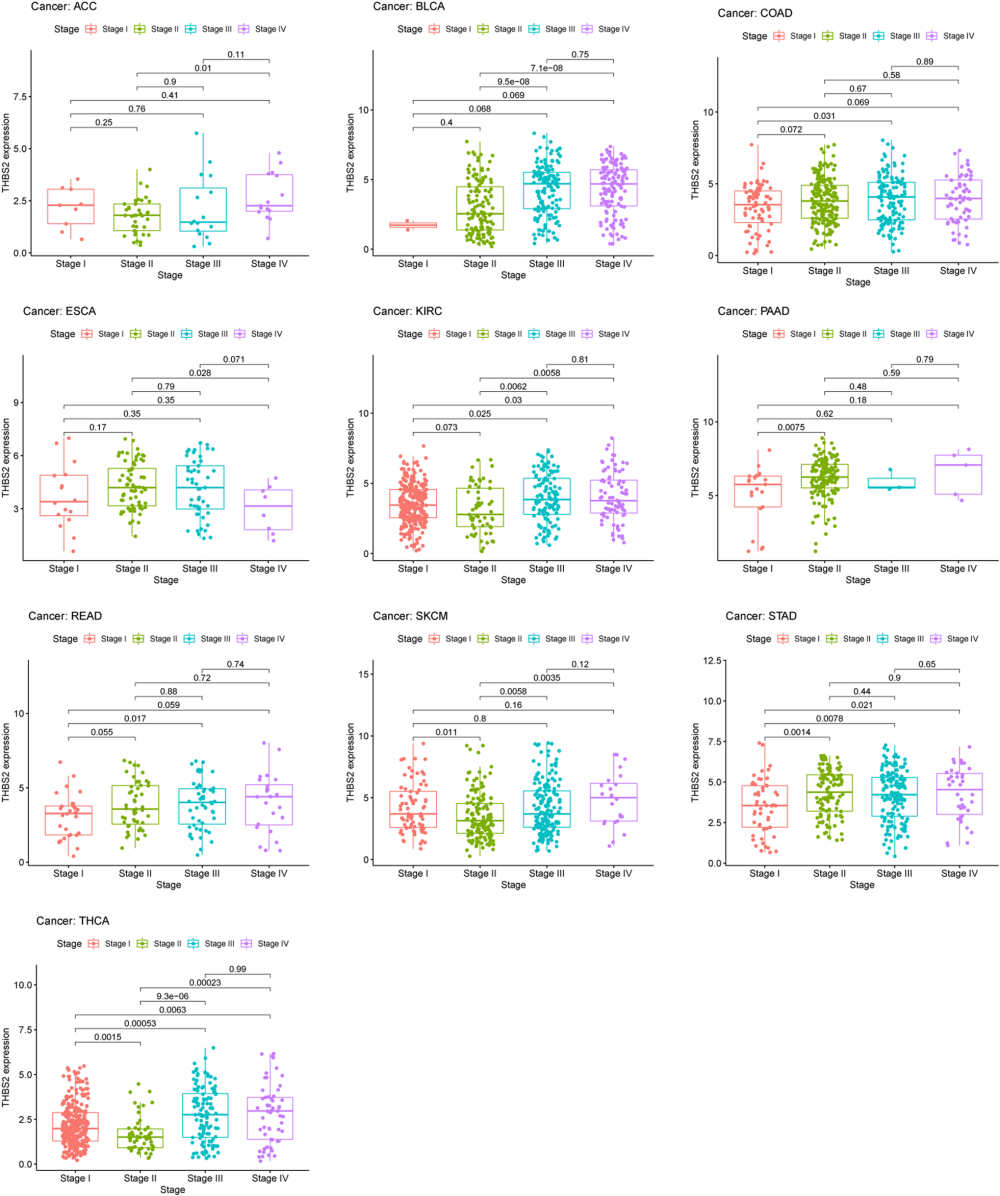
Association between THBS2 expression and stages of 10 types of cancer. Types of cancer: adrenocortical carcinoma (ACC), bladder urothelial carcinoma (BLCA), colon adenocarcinoma (COAD), esophageal carcinoma (ESCA), kidney renal clear cell carcinoma (KIRC), pancreatic adenocarcinoma (PAAD), rectal adenocarcinoma (READ), skin cutaneous melanoma (SKCM), stomach adenocarcinoma (STAD), and thyroid carcinoma (THCA)

**Fig. 7:**
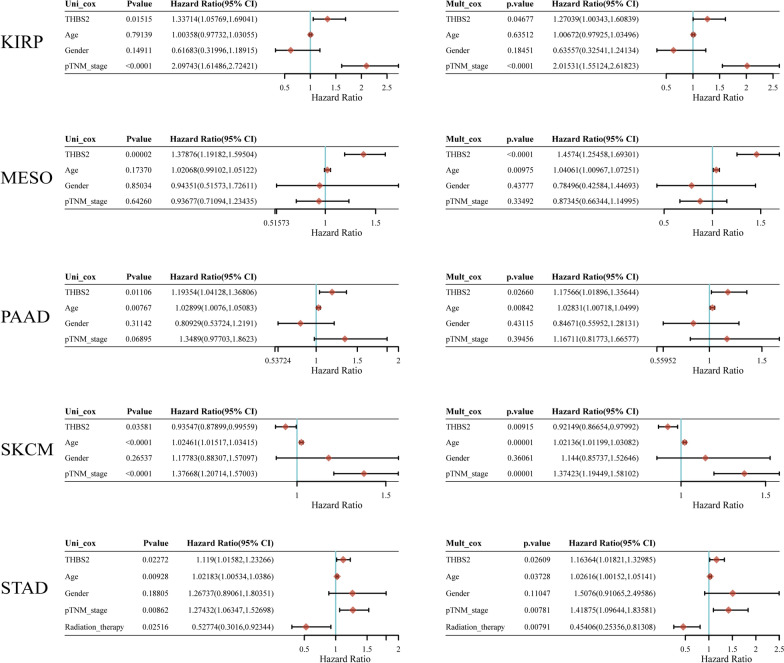
Univariate and multivariate Cox regression analyses of THBS2, tumor pathological staging, and overall survival of kidney renal papillary cell carcinoma (KIRP), mesothelioma (MESO), pancreatic adenocarcinoma (PAAD), skin cutaneous melanoma (SKCM), and stomach adenocarcinoma (STAD) patients

We investigated whether THBS2 expression is associated with patient prognosis in pan-cancer analysis (OS, DSS, DFI, and PFI). We found that THBS2 expression was significantly associated with OS in ACC, BLCA, KIRC, KIRP, LGG, MESO, PAAD, STAD, SKCM, and UVM. (Fig. 2a). Abundant THBS2 expression was associated with a worse OS in all of these types of cancer except SKCM (Fig. 2b–j). The expression of THBS2 influenced DSS in ACC, KIRC, KIRP, LGG, MESO, PAAD, and UVM among 32 types of cancer (Fig. 3a). Kaplan–Meier curves associated increased THBS2 expression with a poor prognosis for these seven types of cancer. (Fig. 3b–h). Forest plots showed that THBS2 expression influenced DFI in ACC, CESC, LIHC, and PAAD (Fig. 4a). Kaplan–Meier curves of DFI associated high THBS2 expression with a worse prognosis in CESC and PAAD (Fig. 4b–c). We assessed the correlations between THBS2 expression and PFI, revealing that THBS2 expression influenced PFI in BRCA, COAD, DLBC, KICH, KIRC, MESO, PAAD, PRAD, and UVM (Fig. 5a). Except for DLBC, increased THBS2 expression negatively impacted PFI in these cancer types. (Fig. 5b–i). Our findings indicated an association between THBS2 and the stages of ACC, BLCA, COAD, ESCA, KIRC, PAAD, READ, SKCM, STAD, and THCA (Fig. 6). More importantly, univariate and multivariate Cox regression analyses were performed to investigate whether THBS2 is an independent prognostic factor for patients with tumors. As shown in Fig. 7, in KIRP, MESO, PAAD, and STAD, results showed that high THBS2 expression was associated with poor OS of patients, indicating that high THBS2 expression was an independent unfavorable prognostic factor in these tumors. In contrast, in SKCM, low THBS2 expression was an independent unfavorable prognostic factor. These findings show that THBS2 expression is substantially linked to patient prognosis in various cancers, particularly MESO, KIRP, STAD, SKCM, and PAAD.

### Expression of THBS2 is correlated with immune infiltration and immune checkpoint markers

**Fig. 8:**
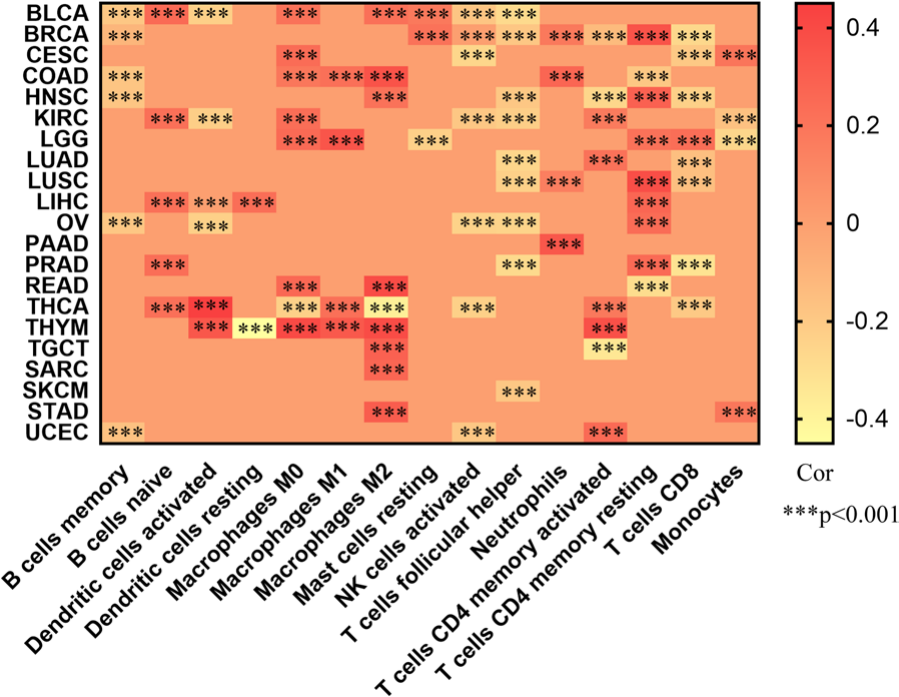
Correlations between THBS2 expression and abundance of infiltrating immune cells among types of cancer

Immune cells play important roles in tumorigenesis and cancer development [15] and are closely associated with patient survival and prognosis [16]. Therefore, we obtained the scores for 15 types of infiltrative immune cells in 21 types of cancer using R. The results are shown as heat maps (Fig. 8). THBS2 expression was significantly correlated with the infiltration levels of B cells in 10 cancer types, dendritic cells in 6 cancer types, macrophages in 12 cancer types, neutrophils in 4 cancer types, CD4 + T cells in 17 cancer types, and CD8 + T cells in 8 cancer types. Moreover, M2 macrophages, T follicular helper cells (Tfh cells), and resting CD4 memory T cells were the three immune cell types most strongly correlated with THBS2 expression across 21 cancer types. Macrophages are the most abundant infiltrative immune-associated stromal cells present in the TME [17], and M2 macrophages primarily promote tumor growth and influence metastasis in various cancers by interacting with cancer cells [18]. We found that M2 macrophages were positively correlated with THBS2 expression in BLCA, COAD, HNSC, READ, THYM, TGCT, SARC, and STAD. Tfh cells, which are required for B-cell maturation and antibody production, have been shown to enhance anti-tumor immune responses, and high levels of infiltrating Tfh cells in the TME are positively associated with better prognosis in patients [19, 20]. Likewise, activated natural killer (NK) cells generally inhibit tumor progression [21]. Accordingly, we found that activated NK cells were negatively correlated with THBS2 expression in BLCA, CESC, KIRC, OV, THCA, and UCEC, and Tfh cells were negatively correlated with THBS2 expression in BLCA, BRCA, HNSC, KIRC, LUAD, LUSC, OV, PRAD, and SKCM. Moreover, THBS2 expression in PAAD was positively correlated with neutrophil infiltration and M2 macrophage and monocyte infiltration in STAD. Overall, in the TME, THBS2 expression was positively correlated with tumor-promoting immune infiltrating cells and negatively correlated with tumor-suppressing immune infiltrating cells. These results suggest that THBS2 plays an important role in mediating tumor immune cells (Fig. 8).

**Fig. 9:**
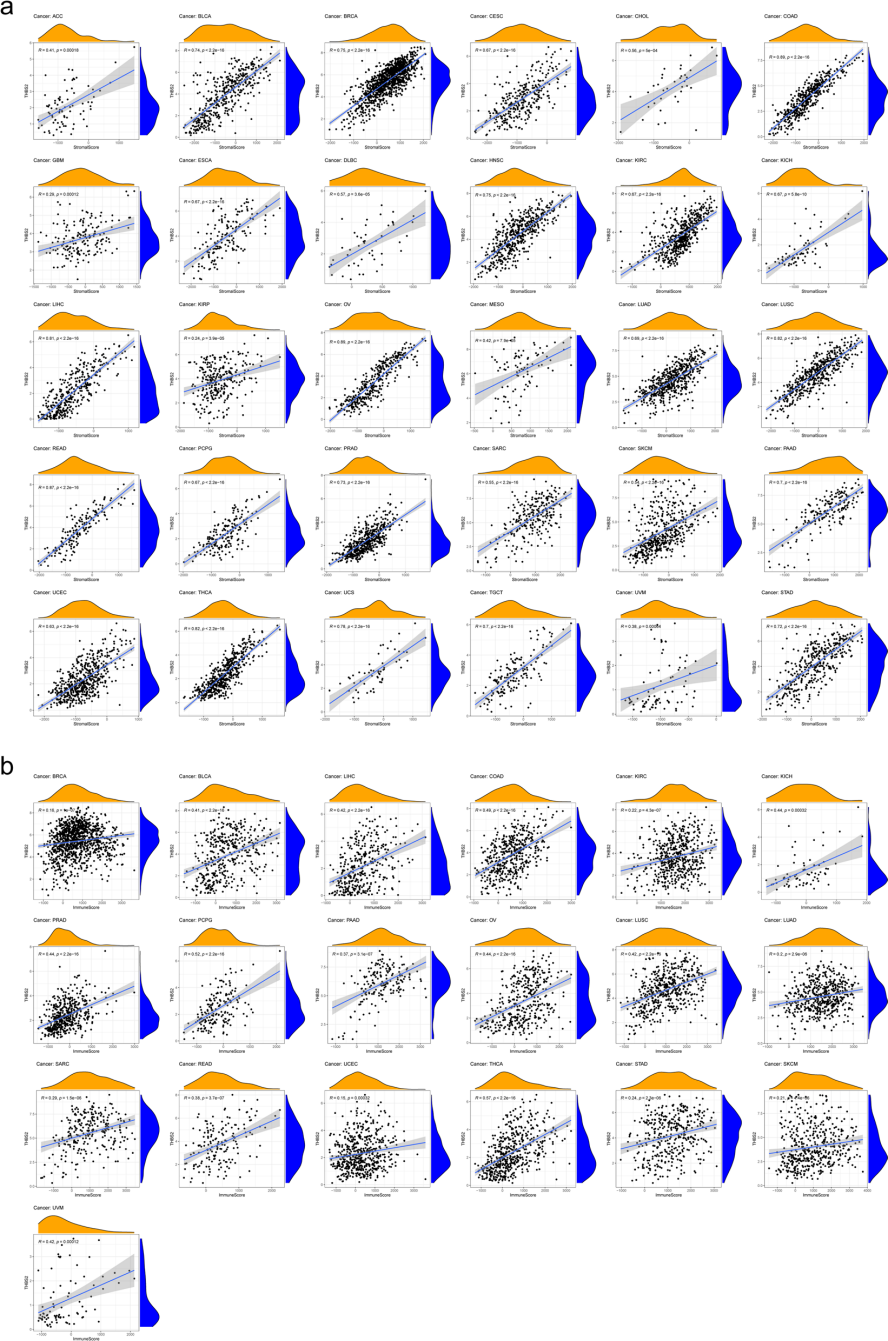
Correlations between THBS2 expression and ImmuneScore/StromalScore in cancers. **a** Correlation between THBS2 expression and ImmuneScore was identified in adrenocortical carcinoma (ACC), bladder urothelial carcinoma (BLCA), breast invasive carcinoma (BRCA), cervical squamous cell carcinoma and endocervical adenocarcinoma (CESC), cholangiocarcinoma (CHOL), colon adenocarcinoma (COAD), glioblastoma multiforme (GBM), esophageal carcinoma (ESCA), lymphoid neoplasm diffuse large B-cell lymphoma (DLBC), head and neck squamous cell carcinoma (HNSC), kidney renal clear cell carcinoma (KIRC), kidney chromophobe (KICH), liver hepatocellular carcinoma (LIHC), kidney renal papillary cell carcinoma (KIRP), ovarian serous cystadenocarcinoma (OV), mesothelioma (MESO), lung adenocarcinoma (LUAD), lung squamous cell carcinoma (LUSC), rectal adenocarcinoma (READ), pheochromocytoma and paraganglioma (PCPG), prostate adenocarcinoma (PRAD), sarcoma (SARC), skin cutaneous melanoma (SKCM), pancreatic adenocarcinoma (PAAD), uterine corpus endometrial carcinoma (UCEC), thyroid carcinoma (THCA), uterine carcinosarcoma (UCS), testicular germ cell tumors (TGCT), uveal melanoma (UVM), and stomach adenocarcinoma (STAD). **b** Correlations between THBS2 expression and StromalScore were identified in BLCA, BRCA, LIHC, COAD, KIRC, KICH, PRAD, PCPG, PAAD, OV, LUSC, LUAD, SARC, READ, UCEC, THCA, STAD, SKCM, and UVM

In addition to analyzing immune-infiltrating cells, we also used ImmuneScore and StromalScore to assess immune and matrix components in pan-cancer analysis. The results indicated that THBS2 expression significantly and positively correlated with the StromalScore in ACC, BLCA, BRCA, CESC, CHOL, COAD, GBM, ESCA, DLBC, HNSC, KIRC, KICH, LIHC, KIRP, OV, MESO, LUAD, LUSC, READ, PCPG, PRAD, SARC, SKCM, PAAD, UCEC, THCA, UCS, TGCT, UVM*,* and STAD (Fig. 9a). THBS2 expression also significantly and positively correlated with ImmuneScore in BRCA, BLCA, LIHC, COAD, KIRC, KICH, PRAD, PCPG, PAAD, OV LUSC, LUAD, SARC, READ, UCEC, THCA, STAD, SKCM, and UVM (Fig. 9b).

**Fig. 10:**
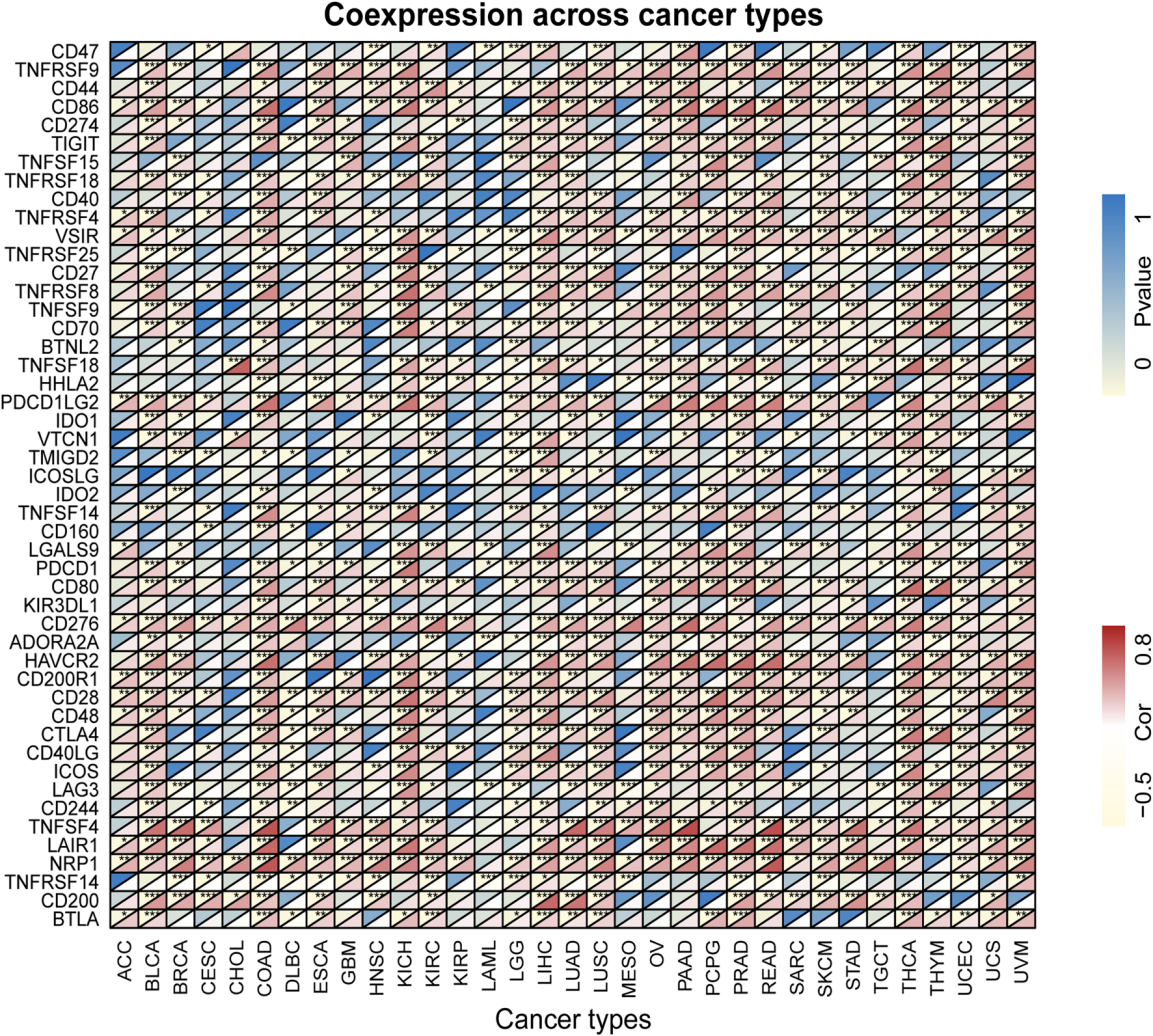
Correlations between THBS2 expression and 48 common immune checkpoint genes in 33 types of cancer. *P < 0.05, **P < 0.01, ***P < 0.001

Given that we identified a relationship between THBS2 expression and immune infiltration, we further analyzed correlations between THBS2 expression and 48 common immune checkpoint genes in 33 cancers. The results indicated that THBS2 expression in BLCA, BRCA, COAD, ESCA, KICH, KIRC, LGG, LIHC, LUAD, LUSC, OV, PAAD, PRAD, PCPG, READ, SKCM, THCA, THYM, UCEC, and UVM correlated with > 30 immune checkpoint markers (Fig. 10). Among these, THBS2 expression was correlated with 45, 42, and 41 immune checkpoint genes in PRAD, COAD, and LIHC and THCA, respectively. These results indicated that THBS2 plays significant roles in tumor immunity and the matrix as an ECM protein.

### Protein–protein interaction (PPI) network of THBS2 in cancer

**Fig. 11:**
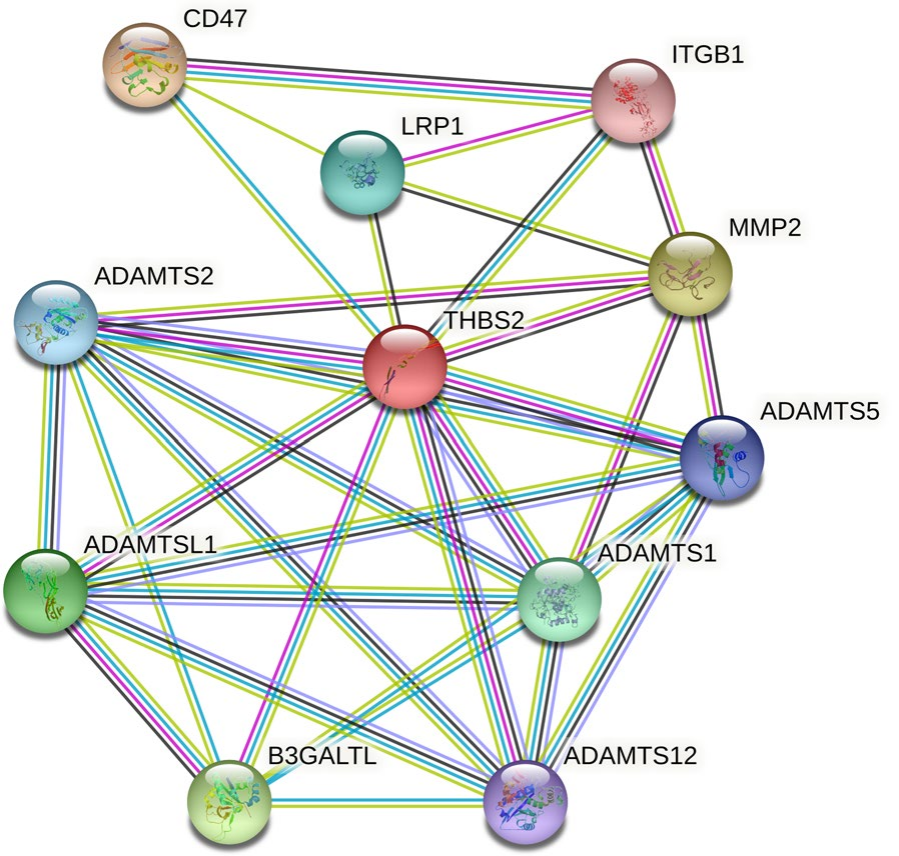
Protein–protein interaction network for THBS2

We created a PPI network for THBS2 using STRING to identify probable processes by which THBS2 contributes to carcinogenesis. Figure 11 shows that THBS2 is closely associated with ECM proteins such as matrix metallopeptidase 2 (MMP-2) and a disintegrin and metalloproteinase with thrombospondin motif (ADAMTS) family and immune proteins such as CD47.

### THBS2 promotes proliferation and metastasis in PAAD and STAD

**Fig. 12:**
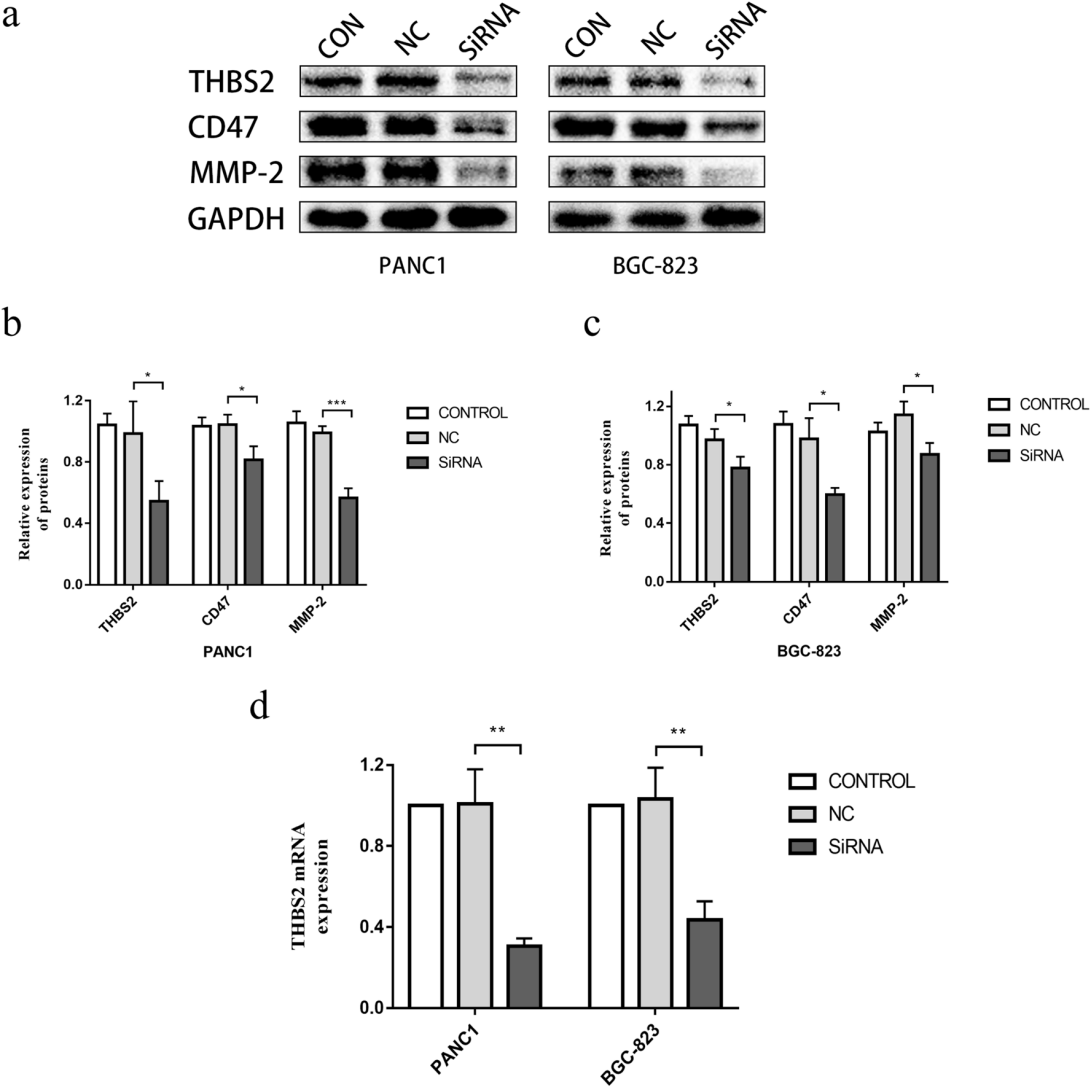
Expression of THBS2 in PANC1 and BGC-823 cells. **a**–**c** Western blots of PANC1 and BGC-823 cells with THBS2 knockdown. **d** Transfection efficiency of THBS2 siRNA in PANC1 and BGC-823 cells validated by qRT-PCR. *P < 0.05, **P < 0.01, ***P < 0.001

**Fig. 13:**
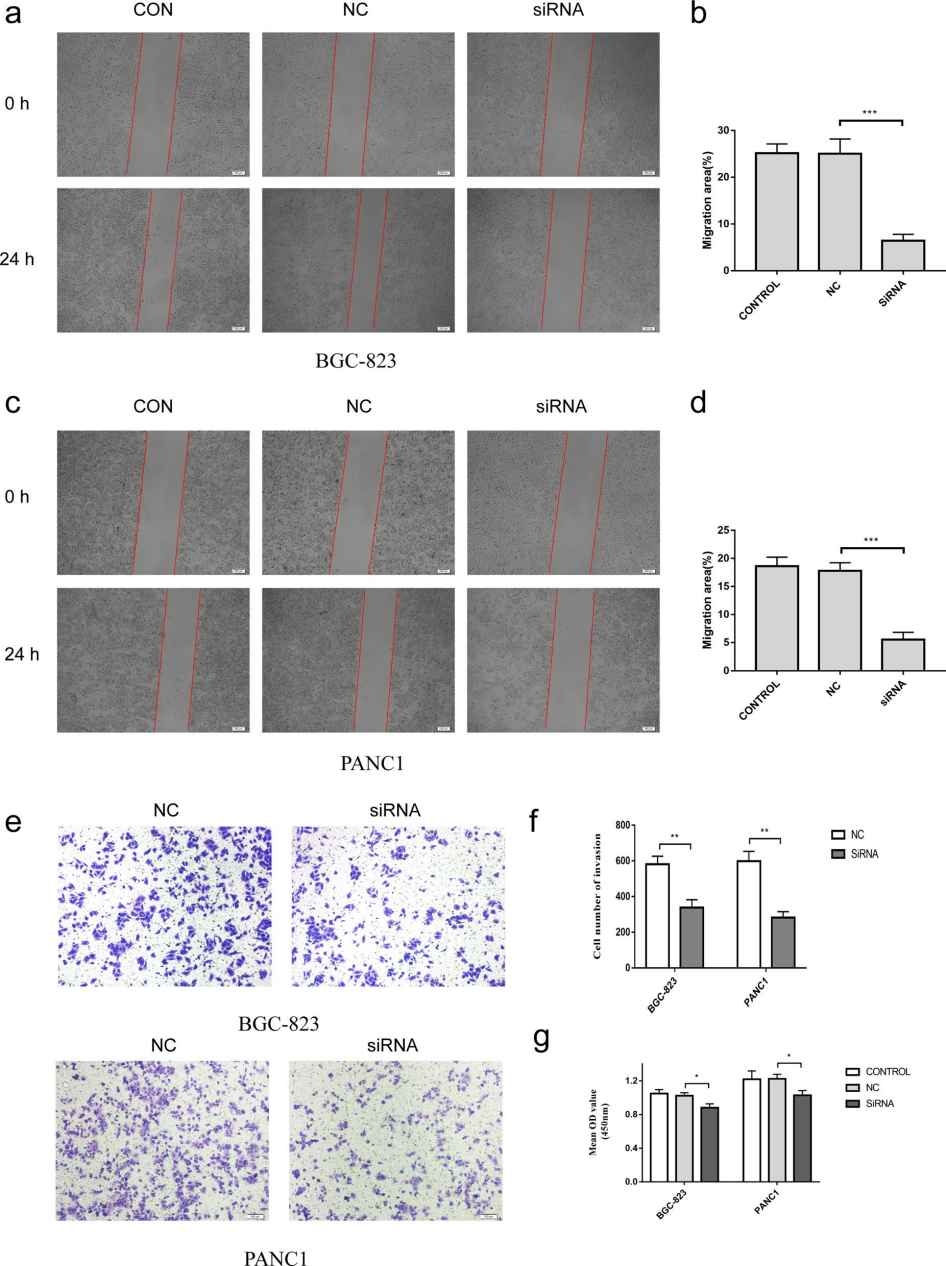
Thrombospondin-2 promotes proliferation and metastasis of PANC1 and BGC-823 cells. **a**–**f** Migration and invasion of PANC1 and BGC-823 cells with THBS2 knockdown. **g** Proliferation of PANC1 and BGC-823 cells with THBS2 knockdown for 24 h. *P < 0.05, **P < 0.01, ***P < 0.001

We performed a pan-cancer analysis of THBS2 expression in 33 tumors, and we found that THBS2 expression was strongly correlated with prognosis in PAAD and STAD. Especially in PAAD, THBS2 expression was significantly associated with poor OS, DSS, DFI, and PFI. In addition, univariate and multifactorial Cox regression analyses confirmed that high THBS2 expression was an independent unfavorable prognostic factor in STAD and PAAD. Moreover, we found that THBS2 was positively correlated with the immune and matrix components of STAD and PAAD. To verify our results and better understand the biological effects of THBS2 on PAAD and STAD, we knocked down THBS2 by transfecting PANC-1 and BGC-823 cells with THBS2 siRNA. Western blotting results showed decreased expression of CD47, MMP-2, and THBS2 (Fig. 12a–c). In addition, CCK8 assay results showed that THBS2 downregulation inhibited PANC1 and BGC-823 cell proliferation (Fig. 13g). Finally, transwell and wound healing assays showed that THBS2 knockdown also inhibited the metastasis and invasion of PANC1 and BGC-823 cells. (Fig. 13a–f). These results indicated that THBS2 promotes the growth and metastasis of PAAD and STAD.

## Discussion

Pan-cancer analysis has recently attracted increasing interest because it can easily and economically analyze tumor similarities and differences, thus revealing potential targets for cancer prevention and treatment [22]. The THBS2 protein belongs to the THBS family and is considered a vital regulator of tumorigenesis. A clinical study of patients with cancer in China has found significant heterogeneity in the distribution of serum THBS2 in diverse types of cancer compared with healthy control individuals [23]. THBS2 is a potential diagnostic marker for pancreatic, gastric, non-small-cell lung, and colorectal cancers [2, 3, 24–26].

Our analysis of TCGA and GTEx database data revealed the abnormal expression of THBS2 in 17 types of cancer relative to that in healthy tissues. Therefore, we further investigated the importance of THBS2 expression in patient prognosis and found that increased THBS2 expression was associated with a worse prognosis (OS, DSS, DFI, and PFI), especially in patients with ACC, KICH, KIRP, PAAD, and STAD. These results indicated that THBS2 could be a prognostic marker for ACC, KICH, KIRP, PAAD, and STAD.

The TME has become a prominent and important area in tumor research. The TME consists of immune and stromal cells, blood vessels, and the ECM [27]. Immune cells comprise infiltrating cells such as B, T, and other types of immune cells, which play dual roles in tumors. On the one hand, they can detect and kill tumor cells [27, 28], whereas, on the other hand, they can allow tumor cells to evade immune surveillance and metastasize via various mechanisms [29–31]. For example, CD8 + T cells can recognize and kill cancer cells that express aberrant tumor antigens. In response to the actions of various cytokines and environments, CD4 + T cells can differentiate into various cell subtypes and participate in the adaptive immune response. Although the TME contains fewer B than T cells, B cells are important for tumor development [32]. In addition to immune-infiltrating cells, other immune cell types have specific functions in the TME. For example, elevated levels of macrophage infiltration are related to a poor prognosis among patients with breast, lung, and gastric cancers [11, 33, 34]. Neutrophils are recruited to the TME early in tumor development and promote tumor cell apoptosis by releasing cytokines and ROS. However, neutrophils can later stimulate angiogenesis by producing MMPs that ultimately promote tumor progression and local invasion, consequently enhancing tumor growth [35]. We found that THBS2 expression in PAAD was positively correlated with neutrophil infiltration and M2 macrophage and monocyte infiltration in STAD. Neutrophils have an immunosuppressive capacity, and tumor neutrophils may achieve a more immunosuppressive state by transitioning to oxidative metabolism [36, 37]. Moreover, tumor neutrophil infiltration is related to metastatic potential in PDAC [38]. Neutrophil extracellular traps release neutrophil elastase and MMP-9, which induce the proliferation of latent metastatic cancer cells and lead to metastatic tumor formation [39]. M2 macrophages, on the one hand, produce IL-10 and TGF-β, leading to suppression of the general anti-tumor immune response. On the other hand, they promote tumor neoangiogenesis by secreting pro-angiogenic factors and establishing the invasive TME to promote tumor metastasis and dissemination [40]. In gastric cancer, IL-6 induces the differentiation of M2 macrophages and promotes the secretion of tumor-promoting cytokines, such as IL-10 and TGF-β, by M2 macrophages, thus facilitating tumor growth and tumor metastasis [18, 41]. Monocytes can be recruited to the TME for differentiation, and in general, TME-infiltrating monocyte-derived cells can be divided into three main subsets: TAM, tumor-associated dendritic cells, and myeloid-derived suppressor cells [42]. Tumor-infiltrating monocyte-derived cells support tumor growth through immunologic and non-immunologic mechanisms. Altogether, immune cells play crucial roles in tumorigenesis and progression. The present study found that THBS2 expression was significantly related to the abundance of various immune cells in various tumor types. We also demonstrated that THBS2 expression is positively correlated with tumor-promoting immune infiltrating cells such as M2 macrophages and negatively correlated with tumor-suppressing immune infiltrating cells, including Tfh and activated NK cells, and is associated with > 30 immune checkpoint genes in 20 cancer types. ImmuneScore and StromalScore are associated with the amount of immune and matrix components in the TME [43]. Our results showed that THBS2 expression positively correlated with ImmuneScore and StromalScore in 30 and 19 cancer types, respectively.

The ECM plays an important role in the TME. In addition to being a physical scaffold for cells, it is also a vital element in driving tumor cell spread. Matrix metalloproteinases comprise a family of zinc- and calcium-dependent proteases that digest ECM proteins and are essential for reshaping the ECM [32]. Furthermore, MMPs not only degrade ECM but also play crucial roles in mediating tumor angiogenesis, metastasis, and invasion. Among the MMP family, MMP-2 promotes tumor growth, tissue invasion, angiogenesis, and metastasis [44–46], and its overexpression might be associated with tumor progression and a poor prognosis for ovarian epithelial carcinoma, oral cavity cancers, and non-small cell lung cancer patients [47–50]. Therefore, MMP-2 is considered a potential tumorigenesis biomarker, a key effector of ECM remodeling, and a potential target for antitumor therapy. A disintegrin and metalloproteinase with thrombospondin motifs (ADAMTSs) interact with various ECM components or regulators to affect cell adhesion, migration, proliferation, and angiogenesis. Furthermore, ADAMTSs can alter the TME through various pathways and exert pro-tumorigenic or anti-tumor properties depending on the substrates or interacting partners present in the microenvironment [51]. CD47, a transmembrane glycoprotein with five transmembrane domains, belongs to the immunoglobulin superfamily and is widely expressed on the surface of almost all normal cells. CD47 negatively regulates anti-tumor immunity by inhibiting phagocytosis, and its overexpression has been observed in most cancers. Moreover, high CD47 mRNA expression levels have been associated with poorer clinical outcomes [52]. Pan et al. showed that CD47 targeting induced compartmental remodeling of tumor-infiltrating immune cells in the TME in pancreatic cancer, and Shi et al. showed that CD47 expression in gastric cancer correlated with macrophage infiltration [53]. In addition, therapies that block the CD47/SIRPα axis may stimulate phagocytosis of cancer cells in vitro and anti-tumor immune responses in vivo through macrophages and other immune cells. Its early clinical trials are also underway [54]. Through PPI networking, we showed that THBS2 in the TME was significantly associated with ECM proteins, including MMP-2, some ADAMTS protein family proteins, and immune-related proteins such as CD47. Finally, our in vitro findings confirmed that THBS2 knockdown inhibited the proliferation, migration, and invasion of PANC1 and BGC-823 cells and decreased the expression of the immune-related protein CD47 and the ECM proteinase MMP-2. These results suggest that THBS2 may act as a bridge between the ECM and immune infiltration, affecting the CD47-mediated signaling pathway, activating the tumor-promoting function of ADAMTs, and enhancing MMP-2 expression, thus remodeling the TME and promoting tumor proliferation and migration (Additional file 1).

## Conclusions

Overall, our findings indicated that THBS2 overexpression correlates with a poor prognosis and increased immune cell infiltration in numerous cancer types. In addition, THBS2 expression positively correlated with tumor-promoting immune infiltrating cells and negatively correlated with tumor-suppressing immune infiltrating cells. Furthermore, THBS2 was closely associated with various ECM and immune proteins and the expression of immune checkpoint markers in various cancer types. Our in vitro results confirmed that THBS2 knockdown inhibited CD47 and MMP-2 expression and the progression of pancreatic and gastric cancers. Thus, THBS2 might promote cancer progression by remodeling the TME, affecting CD47-mediated signaling pathways, activating the pro-tumor functions of ADAMTSs, and enhancing MMP-2 expression. Moreover, THBS2 may function as a bridge between the ECM and immune infiltration in cancer and serve as a potential prognostic biomarker for several cancers, especially pancreatic and gastric adenocarcinomas.

## Supplementary Information

**Additional file 1.** Orignial Images for Western blot

## Data Availability

The datasets used and analyzed during the current study are available from the corresponding author on reasonable request.

## Abbreviations

DFI: Disease-free interval
DSS: Disease-specific survival
ECM: Extracellular matrix
GEPIA2: Gene expression profiling interactive analysis
GTEx: Genotype-tissue expression
MMPs: Matrix metalloproteinases
OS: Overall survival
PFI: Progression-free interval
TAMs: Tumor-associated-macrophages
THBS2: Thrombospondin-2
TIMER2: Tumor immune estimation resource
TME: Tumor microenvironment
Tfh: T follicular helper cells
ADAMTSs: A disintegrin and metalloproteinase with thrombospondin motifs

## References

[1] Adams JC, Lawler J (2004). The thrombospondins. Int J Biochem Cell Biol.

[2] Berger AW, Schwerdel D, Reinacher-Schick A, Uhl W, Algul H, Friess H (2019). A blood-based multi marker assay supports the differential diagnosis of early-stage pancreatic cancer. Theranostics.

[3] Li L, Dong J, Fu L, Xia X, Pan F, Ning Y (2021). clinical value of serum thrombospondin-2 combined with CA19-9 in early diagnosis of gastric cancer. J Oncol.

[4] Wang X, Zhang L, Li H, Sun W, Zhang H, Lai M (2016). THBS2 is a potential prognostic biomarker in colorectal cancer. Sci Rep.

[5] Bindea G, Mlecnik B, Tosolini M, Kirilovsky A, Waldner M, Obenauf AC (2013). Spatiotemporal dynamics of intratumoral immune cells reveal the immune landscape in human cancer. Immunity.

[6] Zhu Y, Yang J, Xu D, Gao XM, Zhang Z, Hsu JL (2019). Disruption of tumour-associated macrophage trafficking by the osteopontin-induced colony-stimulating factor-1 signalling sensitises hepatocellular carcinoma to anti-PD-L1 blockade. Gut.

[7] De Palma M, Biziato D, Petrova TV (2017). Microenvironmental regulation of tumour angiogenesis. Nat Rev Cancer.

[8] Cully M (2018). Cancer: re-educating tumour-associated macrophages with nanoparticles. Nat Rev Drug Discov.

[9] He TF, Yost SE, Frankel PH, Dagis A, Cao Y, Wang R (2020). Multi-panel immunofluorescence analysis of tumor infiltrating lymphocytes in triple negative breast cancer: evolution of tumor immune profiles and patient prognosis. PLoS ONE.

[10] Bocchialini G, Lagrasta C, Madeddu D, Mazzaschi G, Marturano D, Sogni F (2020). Spatial architecture of tumour-infiltrating lymphocytes as a prognostic parameter in resected non-small-cell lung cancer. Eur J Cardiothorac Surg.

[11] Gordon SR, Maute RL, Dulken BW, Hutter G, George BM, McCracken MN (2017). PD-1 expression by tumour-associated macrophages inhibits phagocytosis and tumour immunity. Nature.

[12] Cheng Y, Wang T, Lv X, Li R, Yuan L, Shen J (2020). Detection of PD-L1 expression and its clinical significance in circulating tumor cells from patients with non-small-cell lung cancer. Cancer Manag Res.

[13] Chocarro de Erauso L, Zuazo M, Arasanz H, Bocanegra A, Hernandez C, Fernandez G (2020). Resistance to PD-L1/PD-1 blockade immunotherapy . A tumor-intrinsic or tumor-extrinsic phenomenon. Front Pharmacol.

[14] Newman AM, Steen CB, Liu CL, Gentles AJ, Chaudhuri AA, Scherer F (2019). Determining cell type abundance and expression from bulk tissues with digital cytometry. Nat Biotechnol.

[15] de Visser KE, Eichten A, Coussens LM (2006). Paradoxical roles of the immune system during cancer development. Nat Rev Cancer.

[16] Bonanno L, Pavan A, Dieci MV, Di Liso E, Schiavon M, Comacchio G (2018). The role of immune microenvironment in small-cell lung cancer: distribution of PD-L1 expression and prognostic role of FOXP3-positive tumour infiltrating lymphocytes. Eur J Cancer.

[17] Cheng H, Wang Z, Fu L, Xu T (2019). Macrophage polarization in the development and progression of ovarian cancers: an overview. Front Oncol.

[18] Yamaguchi T, Fushida S, Yamamoto Y, Tsukada T, Kinoshita J, Oyama K (2016). Tumor-associated macrophages of the M2 phenotype contribute to progression in gastric cancer with peritoneal dissemination. Gastric Cancer.

[19] Vinuesa CG, Linterman MA, Yu D, MacLennan IC (2016). Follicular helper T Cells. Annu Rev Immunol.

[20] Lin X, Ye L, Wang X, Liao Z, Dong J, Yang Y (2021). Follicular helper T Cells remodel the immune microenvironment of pancreatic cancer via secreting CXCL13 and IL-21. Cancers (Basel).

[21] Wu SY, Fu T, Jiang YZ, Shao ZM (2020). Natural killer cells in cancer biology and therapy. Mol Cancer.

[22] Schaub FX, Dhankani V, Berger AC, Trivedi M, Richardson AB, Shaw R (2018). Pan-cancer alterations of the MYC oncogene and its proximal network across the cancer genome atlas. Cell Syst.

[23] Zou S, Li J, Yan J, Xu J, Lin M, Cao D (2021). Distribution of serum Thrombospondin-2, a novel tumor marker, in general population and cancer patients in China. Clin Chim Acta.

[24] Peng HY, Chang MC, Hu CM, Yang HI, Lee WH, Chang YT (2019). Thrombospondin-2 is a highly specific diagnostic marker and is associated with prognosis in pancreatic cancer. Ann Surg Oncol.

[25] Jiang YM, Yu DL, Hou GX, Jiang JL, Zhou Q, Xu XF (2019). Serum thrombospondin-2 is a candidate diagnosis biomarker for early non-small-cell lung cancer. Biosci Rep.

[26] Fei W, Chen L, Chen J, Shi Q, Zhang L, Liu S (2017). RBP4 and THBS2 are serum biomarkers for diagnosis of colorectal cancer. Oncotarget.

[27] Junttila MR, de Sauvage FJ (2013). Influence of tumour micro-environment heterogeneity on therapeutic response. Nature.

[28] Morvan MG, Lanier LL (2016). NK cells and cancer: you can teach innate cells new tricks. Nat Rev Cancer.

[29] Dunn GP, Bruce AT, Ikeda H, Old LJ, Schreiber RD (2002). Cancer immunoediting: from immunosurveillance to tumor escape. Nat Immunol.

[30] Tang F, Xu Y, Zhao B (2020). NLRC5: new cancer buster?. Mol Biol Rep.

[31] Simiczyjew A, Dratkiewicz E, Mazurkiewicz J, Zietek M, Matkowski R, Nowak D (2020). The Influence of tumor microenvironment on immune escape of melanoma. Int J Mol Sci.

[32] Anderson NM, Simon MC (2020). The tumor microenvironment. Curr Biol.

[33] Zhang H, Li R, Cao Y, Gu Y, Lin C, Liu X (2020). Poor clinical outcomes and immunoevasive contexture in intratumoral IL-10-producing macrophages enriched gastric cancer patients. Ann Sur.

[34] Choi J, Gyamfi J, Jang H, Koo JS (2018). The role of tumor-associated macrophage in breast cancer biology. Histol Histopathol.

[35] Jaillon S, Ponzetta A, Di Mitri D, Santoni A, Bonecchi R, Mantovani A (2020). Neutrophil diversity and plasticity in tumour progression and therapy. Nat Rev Cancer.

[36] Rice CM, Davies LC, Subleski JJ, Maio N, Gonzalez-Cotto M, Andrews C (2018). Tumour-elicited neutrophils engage mitochondrial metabolism to circumvent nutrient limitations and maintain immune suppression. Nat Commun.

[37] Wang X, Hu LP, Qin WT, Yang Q, Chen DY, Li Q (2021). Identification of a subset of immunosuppressive P2RX1-negative neutrophils in pancreatic cancer liver metastasis. Nat Commun.

[38] Felix K, Gaida MM (2016). Neutrophil-derived proteases in the microenvironment of pancreatic cancer -active players in tumor progression. Int J Biol Sci.

[39] Albrengues J, Shields MA, Ng D, Park CG, Ambrico A, Poindexter ME (2018). Neutrophil extracellular traps produced during inflammation awaken dormant cancer cells in mice. Science.

[40] Sica A, Larghi P, Mancino A, Rubino L, Porta C, Totaro MG (2008). Macrophage polarization in tumour progression. Semin Cancer Biol.

[41] Fu XL, Duan W, Su CY, Mao FY, Lv YP, Teng YS (2017). Interleukin 6 induces M2 macrophage differentiation by STAT3 activation that correlates with gastric cancer progression. Cancer Immunol Immunother.

[42] Ugel S, Canè S, De Sanctis F, Bronte V (2021). Monocytes in the tumor microenvironment. Annu Rev Pathol.

[43] Bi KW, Wei XG, Qin XX, Li B (2020). BTK Has potential to be a prognostic factor for lung adenocarcinoma and an indicator for tumor microenvironment remodeling: a study based on TCGA data mining. Front Oncol.

[44] Chou KY, Chang AC, Ho CY, Tsai TF, Chen HE, Chen PC (2021). Thrombospondin-4 promotes bladder cancer cell migration and invasion via MMP2 production. J Cell Mol Med.

[45] Ni X, Xia T, Zhao Y, Zhou W, Wu N, Liu X (2014). Downregulation of miR-106b induced breast cancer cell invasion and motility in association with overexpression of matrix metalloproteinase 2. Cancer Sci.

[46] Chou KY, Chang AC, Tsai TF, Lin YC, Chen HE, Ho CY (2021). MicroRNA34a5p serves as a tumor suppressor by regulating the cell motility of bladder cancer cells through matrix metalloproteinase2 silencing. Oncol Rep.

[47] Qian Q, Wang Q, Zhan P, Peng L, Wei SZ, Shi Y (2010). The role of matrix metalloproteinase 2 on the survival of patients with non-small cell lung cancer: a systematic review with meta-analysis. Cancer Invest.

[48] Hoffmann C, Vacher S, Sirven P, Lecerf C, Massenet L, Moreira A (2020). MMP2 as an independent prognostic stratifier in oral cavity cancers. Oncoimmunology.

[49] Shi Y, Su C, Hu H, Yan H, Li W, Chen G (2018). Serum MMP-2 as a potential predictive marker for papillary thyroid carcinoma. PLoS ONE.

[50] Jia H, Zhang Q, Liu F, Zhou D (2017). Prognostic value of MMP-2 for patients with ovarian epithelial carcinoma: a systematic review and meta-analysis. Arch Gynecol Obstet.

[51] Cal S, López-Otín C (2015). ADAMTS proteases and cancer. Matrix Biol.

[52] Burugu S, Dancsok AR, Nielsen TO (2018). Emerging targets in cancer immunotherapy. Semin Cancer Biol.

[53] Shi M, Gu Y, Jin K, Fang H, Chen Y, Cao Y (2021). CD47 expression in gastric cancer clinical correlates and association with macrophage infiltration. Cancer Immunol Immunother.

[54] Weiskopf K (2017). Cancer immunotherapy targeting the CD47/SIRPα axis. Eur J Cancer.

