# Supplementary figures and images for: Thrombospondin-2 acts as a bridge between tumor extracellular matrix and immune infiltration in pancreatic and stomach adenocarcinomas: an integrative pan-cancer analysis
Source: Cancer Cell Int. 2022 Jun 14;22:213. doi: 10.1186/s12935-022-02622-x (PMC9195477; doi:10.1186/s12935-022-02622-x)

Figure 12a Original Images for Western blot

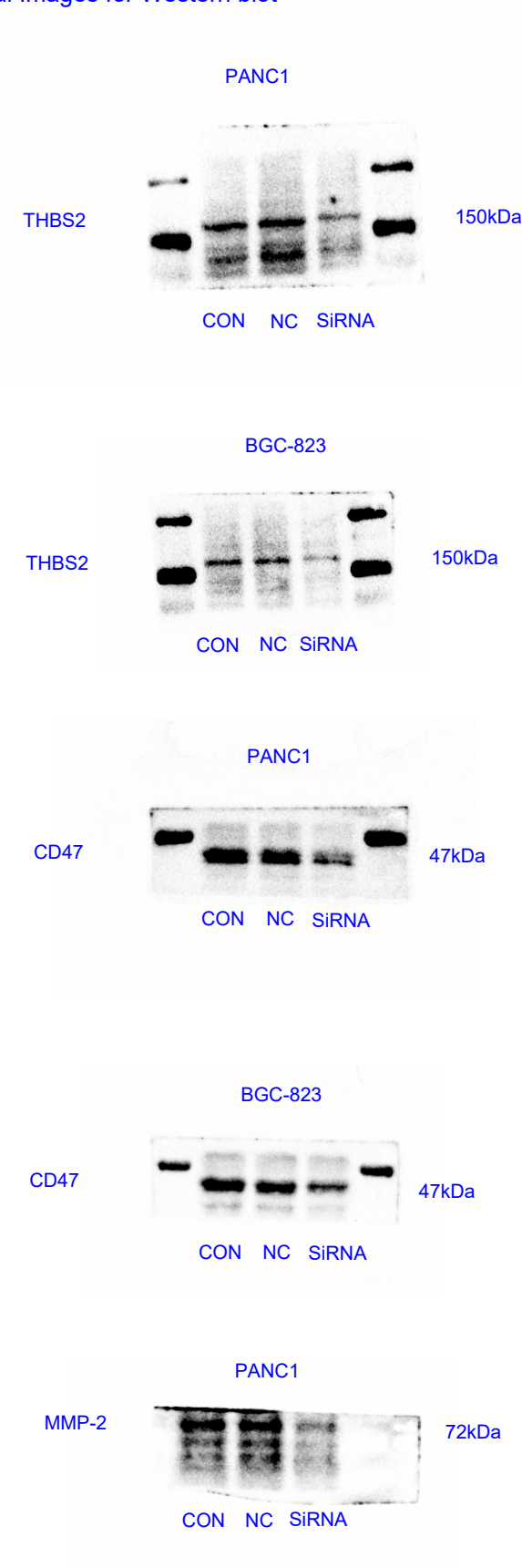

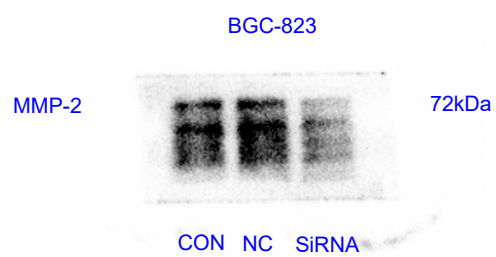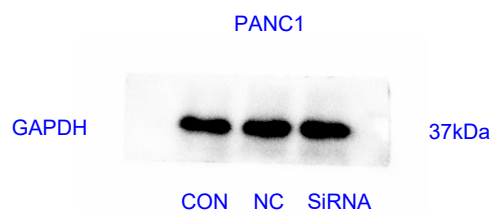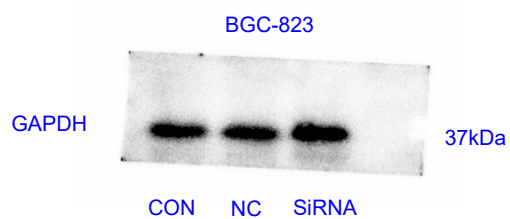

Supplement: Supplementary file 1 — Additional file 1. Orignial Images for Western blot [file 12935_2022_2622_MOESM1_ESM.pdf]
